# Visual function and retinal morphological changes after single suprachoroidal delivery of fluocinolone acetonide (Iluvien®) implant in eyes with chronic diabetic macular edema

**DOI:** 10.1186/s40942-023-00458-9

**Published:** 2023-03-29

**Authors:** Ehab N. El Rayes, Mahmoud Leila

**Affiliations:** Retina department, Research Institute of Ophthalmology (RIO), 35 Salah Salem St., (El Borg), Suite 702, El-Obour bldg., Cairo, 11371 Egypt

**Keywords:** Suprachoroidal Iluvien, Suprachoroidal fluocinolone acetonide, Suprachoroidal steroids, Suprachoroidal drugs for DME

## Abstract

**Background:**

To assess the efficacy and safety of supra-choroidal (SC) Iluvien in the management of chronic diabetic macular edema (DME).

**Methods:**

A retrospective interventional non-comparative consecutive case series including patients with chronic DME who received an SC Iluvien implant. All patients had persistent central macular thickness (CMT) ≥ 300µ after previous treatment with anti-vascular endothelial growth factor (VEGF) agents or laser photocoagulation. The main outcome measures were improvement of best-corrected visual acuity (BCVA), reduction of CMT, and detection of ocular hypertension/glaucoma or cataract formation. Friedman’s two-way ANOVA was used to analyze BCVA, intraocular pressure (IOP), and DME across different time points. P-value = 0.05.

**Results:**

The study included 12 eyes of 12 patients. Six patients (50%) were males. The median age was 58 years (range 52–76 years). The median duration of DM was 13 years (range 8–20 years). Ten patients (83.3%) were phakic and 2 patients (17%) were pseudophakic. The median pre-operative BCVA was 0.07 (range 0.05–0.8). The median pre-operative CMT was 544µ (range 354–745µ). The median pre-operative IOP was 17 mmHg (range 14-21mmHg). The median follow-up period was 12 months, range (12–42). Post-operatively, the median final BCVA was 0.15 (range 0.03-1), p 0.02, the median CMT was 404µ (range 213–747), p 0.4 and the median IOP was 19.5 mmHg (range 15–22), p 1. Two out of 10 phakic patients (20%) developed nuclear sclerosis grade I by 12 months. Six patients (50%) developed a transient rise in IOP < 10 mmHg from the baseline that resolved within 3 weeks with antiglaucoma drops.

**Conclusion:**

SC Iluvien is potentially effective in improving visual function, reducing macular edema, and reducing the incidence of steroid-induced cataracts and glaucoma.

**Supplementary Information:**

The online version contains supplementary material available at 10.1186/s40942-023-00458-9.

## Background

Anti-vascular endothelial growth factor (Anti-VEGF) agents are the current standard treatment for diabetic macular edema (DME) [1–4]. Nevertheless, anti-VEGF agents have several limitations. Almost one-third of patients show limited response to anti-VEGF agents despite strictly abiding by the required treatment and follow-up protocols [5]. Anti-VEGF therapeutic regimens require repeated injections over relatively short periods of time to maintain initial favorable anatomic and functional outcomes, hence adversely affecting the quality of life of the patient and increasing the risk of endophthalmitis and retinal detachment [2, 6, 7]. Another challenge is the rapid clearance of the drug in vitrectomized eyes leading to suboptimal response [8]. In the early phases of DME, anti-VEGF agents are highly effective in neutralizing the vasogenic changes mediated by VEGF, which eventually lead to a breakdown of the inner blood-retina-barrier (BRB) and subsequent development of DME. As chronicity ensues, the inflammatory cascade produces several inflammatory mediators that promote leakage of leucocytes into the extracellular tissues, hence aggravating DME. In this latter stage, the efficacy of anti-VEGF agents declines dramatically because many of these inflammatory mediators are not amenable to their therapeutic effect [4, 9, 10]. Triamcinolone acetonide, dexamethasone, and fluocinolone acetonide (Fac) are broad-spectrum anti-inflammatory steroids. These steroids reduce DME by targeting the inflammatory cascade in a multi-pronged approach that includes inhibition of the inflammatory mediators responsible for the breakdown of the BRB, leucocyte migration, and VEGF production, hence, the more comprehensive and effective long-term control of DME compared to anti-VEGF agents. Nevertheless, the intravitreal route of steroid delivery shares the same intra-ocular risks as intravitreal anti-VEGF agents, and triamcinolone acetonide is cleared faster in vitrectomized eyes [11–15]. In addition, intravitreal steroids induce cataracts and glaucoma, which is the main reason they are used as a second-line choice in cases resistant to anti-VEGF [1, 4, 16–19]. Iluvien is a non-biodegradable implant impregnated with 0.19 mg of a synthetic fluorinated glucocorticoid; Fac. The implant releases 0.25 µg/day of Fac [20–22]. Suprachoroidal (SC) delivery of Iluvien circumvents the complications related to intravitreal injection including lens or retina injury and endophthalmitis. In addition, the SC route bypasses the sclera, outer BRB, and internal limiting membrane (ILM) and delivers the drug directly to the posterior pole [23–25]. Fac is a highly lipophilic drug, thus once delivered past these barriers it readily penetrates the choroid and retina and yields therapeutic concentration [22]. Moreover, the SC space is normally confined by the scleral spur, vortex ampullae, and short ciliary vessels, which helps compartmentalization of Fac in the posterior pole and limits its side effect on the anterior segment of the eye particularly cataract formation and glaucoma [23, 26]. The aim of the present study is to assess the efficacy and safety of SC Iluvien in the management of chronic DME.

## Methods

**Fig. 1:**
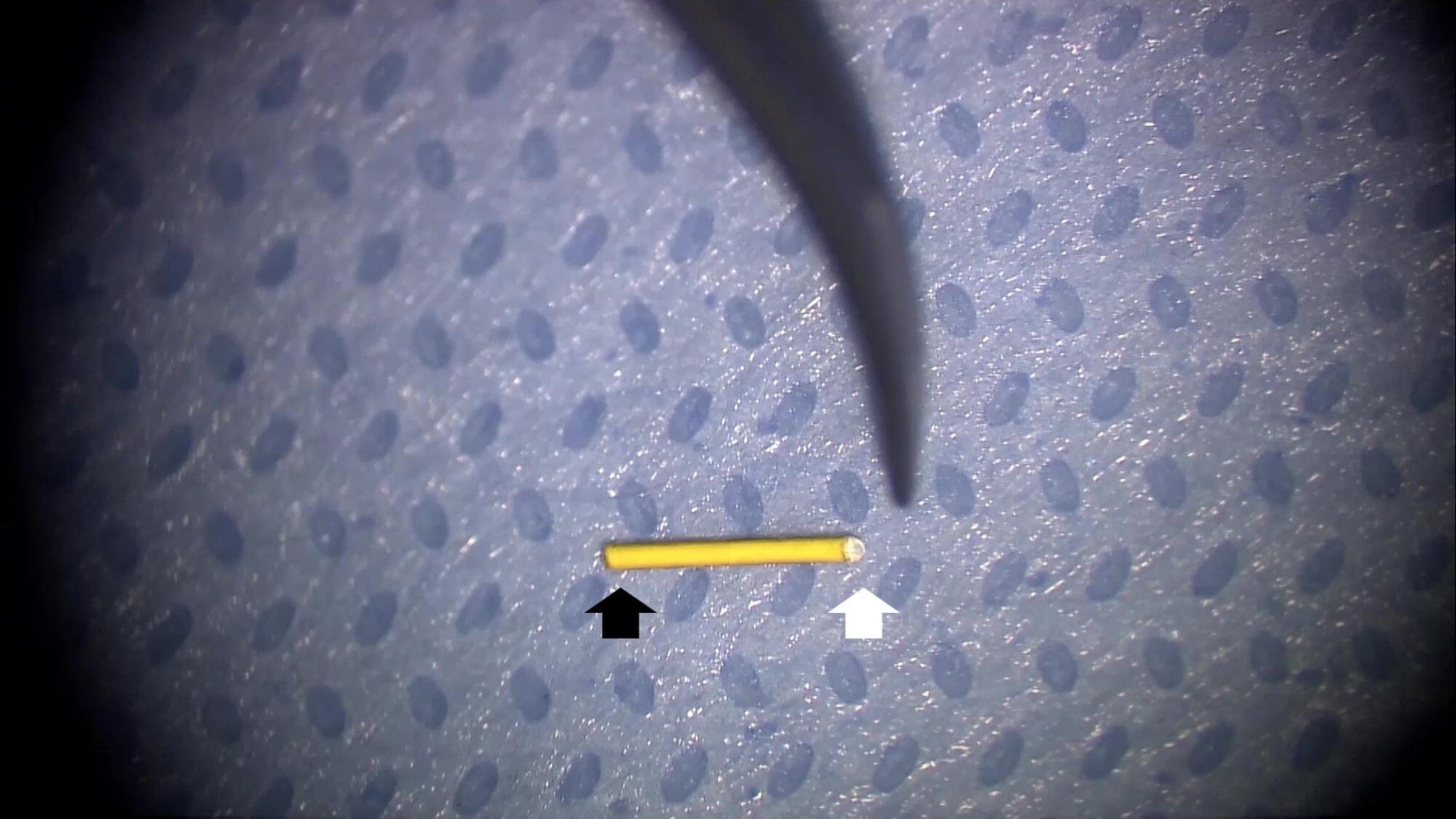
Iluvien implant. The implant is cylindrical, light-brown in color, and measures 3.5 × 0.37 mm. The implant has 2 ends. An active one that releases the drug (white arrow) and a sealed inactive end (black arrow)

**Fig. 2:**
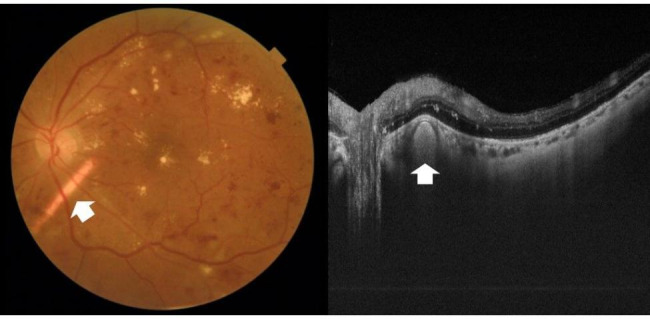
Post-operative color fundus photo and corresponding OCT scan of the left eye of a 58-year-old female patient. The Iluvien implant is located in the SC space inferotemporal to the optic nerve head (white arrows)

This is a retrospective interventional non-comparative consecutive case series including patients with chronic DME who received an SC Iluvien implant. The study was conducted in the retina department of the Research Institute of Ophthalmology (R.I.O.), Egypt between April 2017 and April 2019. Inclusion criteria were diabetic patients with type I or type II diabetes mellitus (DM) and chronic DME. Chronic DME was defined as persistent macular edema with central macular thickness (CMT) on optical coherence tomography (OCT) scan ≥ 300 microns (µ) after previous treatment with at least 3 intravitreal injections of anti-VEGF agents or laser focal or grid photocoagulation. The study required that the last anti-VEGF injection was delivered not less than 2 months and the last laser treatment was delivered not less than 4 months. Exclusion criteria included prior history of glaucoma or ocular hypertension, vitreomacular traction, patients requiring concomitant pan laser photocoagulation for proliferative diabetic retinopathy, concomitant retinal vascular disease, or central macular pathology that could cause neurosensory macular detachment, concomitant optic nerve pathology as diabetic papillopathy, and patients with media opacities that would hinder reliable imaging on OCT or intraoperative visualization and patients with macular ischemia involving ≥ 2 quadrants on fundus fluorescein angiography. The main outcome measures were improvement of baseline best-corrected visual acuity (BCVA), reduction of CMT on OCT, and detection of ocular hypertension/glaucoma or cataract formation secondary to Iluvien use. Recruited patients received full ophthalmological examination including slit-lamp anterior segment examination, intraocular pressure (IOP) assessment using applanation tonometry, and slit-lamp biomicroscopy. BCVA was recorded using decimal notation. OCT imaging was performed using swept-source OCT (DRI OCT Triton 10.11; Topcon, Tokyo, Japan). FFA was performed using Topcon TRC 50DX fundus camera (Topcon Corporation, Tokyo, Japan) when needed. Surgery was performed by a single surgeon (ENE). SC implantation of Iluvien is performed without vitrectomy. Chandelier light is applied through a single port using a 25-g trocar system. A conjunctival incision is performed at 12 o’clock followed by a 2-mm scleral incision placed at 4 mm parallel to the limbus. The last lamella of the sclera is opened using diathermy, taking care not to injure the choroid. A small amount of viscoelastic is injected through the scleral incision to separate the choroid from the sclera and open the SC space for easy introduction of the cannula. The Iluvien implant 0.19 mg is advanced outside its container and reloaded into a flexible tip disposable cannula (Olive Tip SC Cannula, MedOne Surgical) after priming the cannula with viscoelastic. The releasable end of the implant is placed towards the open end of the cannula. The cannula is gradually advanced into the SC space along the scleral lip of the sclerotomy in between the choroid and the sclera while pushing back against the sclera. The Olive Tip dissects the path during the advancement of the cannula into the SC space under direct visualization through the surgical microscope assisted by the chandelier light until reaching the desired location around the vascular arcades. The plunger is then pushed gently to displace the implant by the viscoelastic out of the cannula into the desired location in the SC space. Afterward, the catheter is withdrawn although the scleral incision and the chandelier light is removed. The scleral incision is closed using a single 7/0 vicryl suture. **SDC 1.** Figs. 1 and 2. The post-operative follow-up was performed on the 1st day post-operative, 1 week, 1 month, and then at a 3-monthly visit. The post-operative assessment included BCVA measurement, IOP measurement, evaluation of the implant location using slit-lamp biomicroscopy, and OCT measurement of CMT. The minimum follow-up period was 12 months. The present study adhered strictly to the tenets of the Declaration of Helsinki (2013). The study protocol was approved by the institutional review board of the Research Institute of Ophthalmology. All patients included in the study signed an informed consent after receiving a thorough explanation regarding the surgical procedure entailed in the study, possible outcomes, and expected complications. The consent included a statement that allowed the anonymous publication of patients’ data for research purposes.

### Statistical analysis

Patients’ characteristics were described by frequencies and percentages for categorical variables and medians, minimums, and maximums for continuous data. Correlations between the duration of DM (DMD) versus IOP, BCVA, and DME were analyzed using spearman’s correlation. Friedman’s two-way ANOVA was used to analyze BCVA, IOP, and DME across different time points. Analysis was computed at a significance level of 0.05.

## Results

**Fig. 3:**
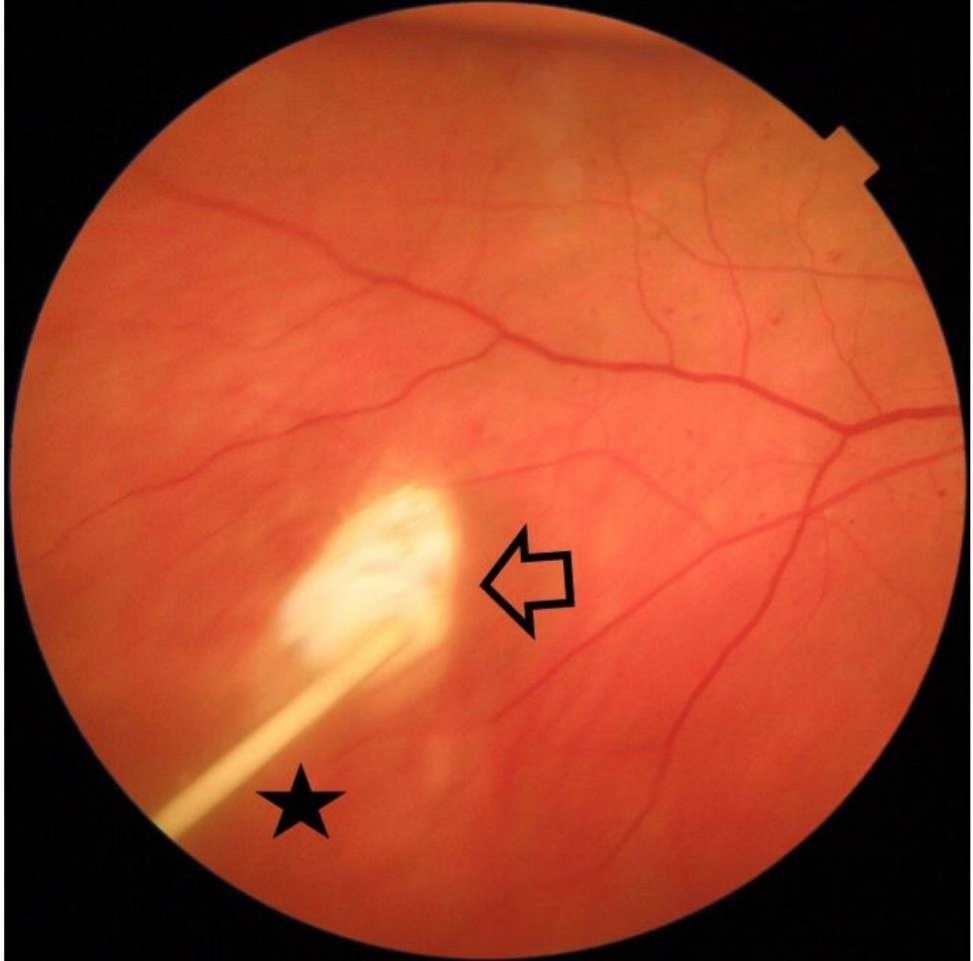
Color fundus photo of the right eye of a 52-year-old male patient showing intravitreal migration of Iluvien implant (black asterisk) after initial SC delivery. Note laser retinopexy marks around the site of retinal perforation (black arrow)

**Fig. 4:**
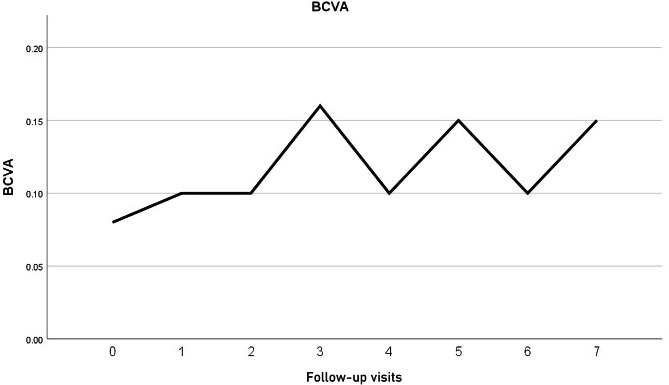
Timeline of BCVA (decimal notation) variation throughout the follow-up period

**Fig. 5:**
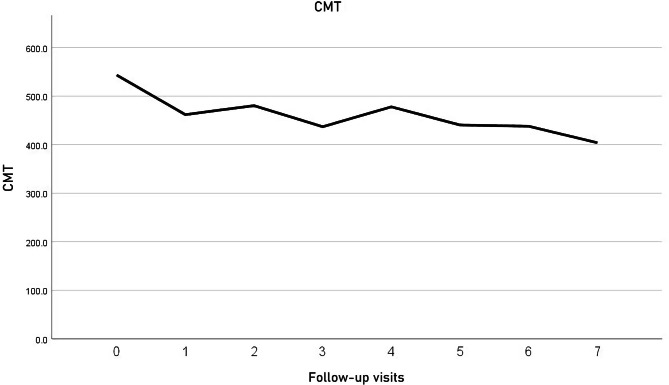
Timeline of CMT (microns) variation throughout the follow-up period

**Fig. 6:**
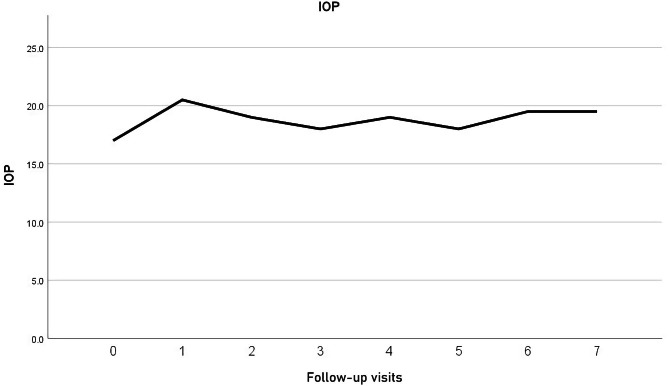
Timeline of IOP variation throughout the follow-up period

**Fig. 7:**
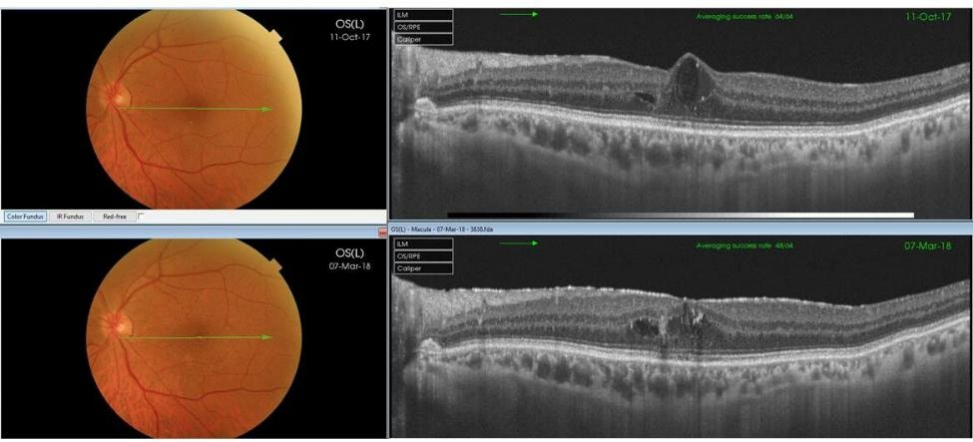
Top. Pre-operative color fundus photo and corresponding OCT scans of the macular area of the left eye of a 52-year-old female patient show spongy retinal edema with a large subfoveal cyst. BCVA was 0.2. Bottom. Corresponding color fundus photo and OCT scan 6 months post-operatively. Note the significant improvement of macula edema and restoration of retinal contour. The final BCVA was 0.5

**Fig. 8:**
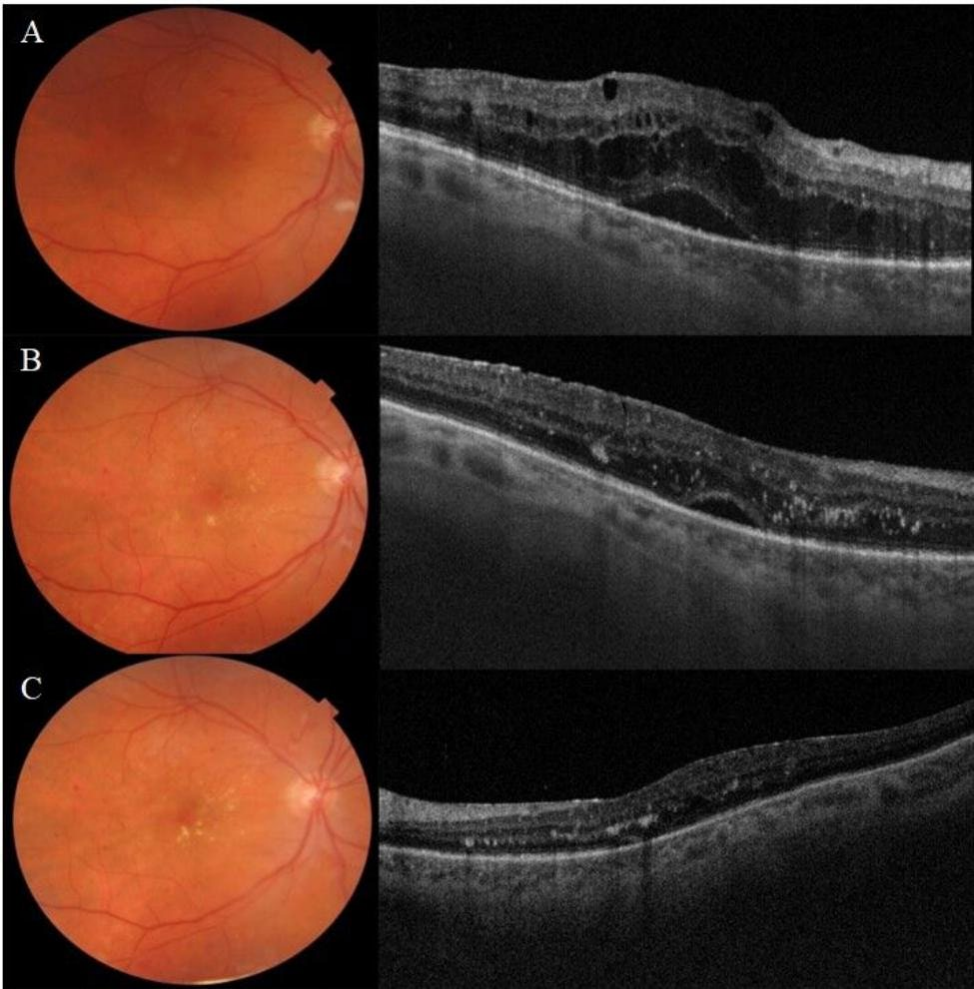
A, B, C. Serial color fundus photos and OCT scans of the macular area of the right eye of a 62-year-old female patient. A. Pre-operatively, the OCT scan shows diffuse macular edema, cystoid spaces, and sub-foveal neurosensory detachment. BCVA was 0.1. B, C. OCT scans of the macula area at 3 & 6 months, respectively. Note progressive resolution of inner retinal edema, neuro-sensory detachment, and restoration of retinal contour. The final BCVA was 0.4

The study included 13 eyes of 13 patients. In one patient the Iluvien implant migrated into the vitreous cavity at 1 month of follow-up. We decided to leave the implant in the vitreous cavity and apply laser retinopexy to the small retinal break in the office. There was no associated retinal detachment and the patient was excluded from the study. Figure 3. All remaining 12 patients completed the minimum required follow-up period. Six patients (50%) were males. The median age was 58 years (range 52–76 years). The median duration of DM was 13 years (range 8–20 years). Ten patients (83.3%) were phakic and 2 patients (17%) were pseudophakic. Two patients (17%) had received previously 6 and 8 intravitreal anti-VEGF injections, respectively. Four patients (33%) had received previously focal/grid laser photocoagulation combined and at least 1 intravitreal anti-VEGF injection. Six patients (50%) had received previously focal/grid laser photocoagulation solely. The median pre-operative BCVA was 0.07 (range 0.05–0.8). Median pre-operative CMT was 544µ (range 354–745µ). The median pre-operative IOP was 17 mmHg (range 14-21mmHg). Table 1. Iluvien implant was placed in the superior quadrant in 9 patients (75%) and in the superotemporal, inferotemporal, and inferior quadrants in one patient (8.3%) each. The Median follow-up period was 12 months, range (12–42). Post-operatively, median final BCVA was 0.15 (range 0.03-1), p 0.02, median final CMT was 404µ (range 213–747), p 0.4 and median IOP was 19.5 mmHg (range 15–22), p 1. Figures 4, 5, 6, 7 and 8. Ten patients (83.3%) were phakic, of which 3 patients (30%) had nuclear sclerosis cataract grade I-II at enrollment. None of these 3 patients had a progression of sclerosis through the end of the follow-up period. Two out of 10 phakic patients (20%) developed nuclear sclerosis grade I by 12 months with stable visual acuity. Six patients (50%) developed a transient rise in IOP during the first post-operative month that required initiation of topical anti-glaucoma drops (combination of b-blocker and carbonic anhydrase inhibitor). The IOP spike in all 6 patients did not exceed 10 mmHg from the baseline or reached ≥ 30 mmHg at any time point. In all 6 patients, IOP returned to the baseline within 3 weeks and anti-glaucoma drops were discontinued Table 2.

**Table 1:** Baseline patients’ characteristics

Baseline Characteristics	N (%)
Male	6 (50)
Female	6 (50)
Age (years)	
˂60 60–65 > 65	7 (58.3) 4 (33.3) 1 (8.3)
Duration of DM (years)	
˂10 10–15 > 15	2 (17) 6 (50) 4 (33.3)
BCVA (decimal)	
˂0.1 0.1–0.5 > 0.5	2 (17) 9 (75) 1 (8.3)
IOP (mmHg)	
˂15 15–20 > 20	1 (8.3) 10 (83.3) 1 (8.3)
CMT (µ)	
< 400 400–600 > 600	3 (25) 5 (41.6) 4 (33.3)
Lens status	
Phakic • Clear lens • Pre-existing sclerosis Pseudophakic	10 (83.3) 7 (70) 3 (30) 2 (17)

BCVA, best-corrected visual acuity; CMT, central macular thickness; DM, diabetes mellitus; IOP, intraocular pressure; µ, micron

**Table 2:** Postoperative outcome

Post-operative outcome	N (%)
Location of Iluvien implant (quadrant)	
Superior Supero-temporal Inferior Infero-temporal	9 (75) 1 (8.3) 1 (8.3) 1 (8.3)
Follow-up (months)	
12 > 12–24 > 24	7 (58.3) 4 (33.3) 1 (8.3)
Final BCVA (decimal)	
˂0.1 0.1–0.5 > 0.5	6 (50) 5 (42) 1 (8.3)
Final CMT (µ)	
< 400 400–600 > 600	6 (50) 4 (33.3) 2 (17)
Final IOP (mmHg)	
˂15 15–20 > 20	0 (0) 10 (83.3) 2 (17)
Complications	
Transient IOP spike Nuclear sclerosis grade I	6 (50) 2 (20)

BCVA, best-corrected visual acuity; CMT, central macular thickness; IOP, intra0ocular pressure; µ, micron

## Discussion

The present study is the first that used the SC approach for delivering Iluvien to patients with chronic DME. The study assessed the efficacy of placing Iluvien in the SC space in improving DME, and whether it reduced the incidence of cataracts and of glaucoma. The results showed that SC Iluvien produced significant improvement of median BCVA by 3 lines and 50% of patients gained ≥ 3 lines of vision. Our results are comparably better than other studies using intravitreal Iluvien for chronic DME, which reported BCVA gain ≥ 15 letters in 28%, and 17% of patients, respectively [27, 28]. Similarly, other authors reported an increase in BCVA in 75% of patients by 3.7 letters in one study and a mean increase in BCVA by the same value in another [29, 30]. In our study, 50% of patients had a final BCVA ≥ 0.1, and 8.3% had a final BCVA > 0.5. In comparison, other studies reported final BCVA > 6/12 in 31–33%, 26–33%, and 31% of patients, respectively [27, 28, 31]. In terms of improvement of macular edema, we had a 25% reduction in median CMT compared to baseline. In comparison, other authors reported 31–34%, and 26% reduction of CMT from baseline [27, 28]. Other studies had a reduction in mean CMT by 203, and 61µ from baseline [29, 30]. It is worthy of note that the majority of the fore-mentioned studies allowed additional treatment using another implant, rescue treatment using another line of therapy for macular edema, or protocol deviation that involved as many as 39% of cases [22, 27, 28, 30, 32, 33]. In comparison, all results of BCVA and CMT in the present study were achieved following a single application of SC Iluvien. In terms of the safety of SC Iluvien, in the present study, 50% had a transient IOP spike that resolved within 3 weeks after surgery with antiglaucoma drops. Subsequently, we discontinued the anti-glaucoma drops, and IOP remained stable through the end of the follow-up. Two out of 10 phakic patients (20%) developed nuclear sclerosis by 12 months with stable visual acuity. There was no progression of pre-existing sclerosis in 3 patients (30%) at the end of the follow-up. In comparison, 74.9-84.5%, 25%, and 62% in the FAME [27], RESPOND [29], and PALADIN [30] trials developed cataract. The FAME study performed laser trabeculoplasty (LTP) and incisional glaucoma surgery to lower the IOP in 0.8–2.3% and 3.7–8.1% of patients, respectively [27]. The PALADIN study performed LTP and glaucoma surgery in 2% and 1.5% of patients, respectively [30]. The IRISS study performed glaucoma surgery in 4.7% of patients [32]. In the Medisoft audit study 2% and 5% of patients with prior IOP – related events had LTP and glaucoma surgery, respectively; whereas 1.2% of patients without prior IOP – related events had glaucoma surgery [28]. Analysis of the fore-mentioned safety data reveals that SC Iluvien is superior to other studies that used intravitreal Iluvien in terms of incidence of glaucoma. It is also superior to other studies in terms of cataractogenesis. Therefore, our results corroborate the concept that the SC location of Iluvien is safer than the intravitreal route of administration due to the sequestration of the drug in the posterior pole remotely from the anterior segment structures [23, 26]. This finding along with the visual and anatomical results of our study indicates that a single application of SC Iluvien is at least equally effective and safer than intravitreal administration. Limitations of the present study include its small sample size and lack of concurrent comparison with other treatment modalities for chronic macular edema.

## Conclusion

The SC route for delivering Iluvien is a promising novel technique that is effective in improving visual function,reducing macular edema, and reducing steroid-induced cataracts and glaucoma in patients with chronic DME. Its efficacy and safety need to be consolidated by prospective comparative studies that include a larger sample size before recommending its use as a standard treatment.

## Electronic supplementary material

Below is the link to the electronic supplementary material.

Supplementary Material 1: **SDC1.** The video shows the surgical technique of SC implantation of Iluvien in several patients

Supplementary Material 2

Supplementary Material 3

## Data Availability

All data pertaining to the present study are confidential. Access to these data will be granted exclusively to people or entities who meet the criteria for access to confidential data and only upon written request. All requests should be addressed to the corresponding author: Professor Ehab N. El Rayes. 35 Salah Salem St., (El Borg), Suite 702, El-Obour bldg. Cairo 11,371, Egypt.
