# Supplementary material for: Visual function and retinal morphological changes after single suprachoroidal delivery of fluocinolone acetonide (Iluvien®) implant in eyes with chronic diabetic macular edema
Source: Int J Retina Vitreous. 2023 Mar 29;9:20. doi: 10.1186/s40942-023-00458-9 (PMC10053734; doi:10.1186/s40942-023-00458-9)
Supplement: Supplementary file 2 — Supplementary Material 2 [file 40942_2023_458_MOESM2_ESM.docx]

**Cover letter**

February 16, 2023

Antonio Marcelo Barbante Casella, MD

Gustavo Barreto de Melo, MD

Editors-in-chief

International Journal of Retina and Vitreous

Dear Sir,

I, herewith, submit our manuscript, entitled: ‘Visual function and retinal morphological changes after single suprachoroidal delivery of fluocinolone acetonide (Iluvien®) implant in eyes with chronic diabetic macular edema’, for consideration for publication in the International Journal of Retina and Vitreous. Our work is a retrospective interventional non-comparative consecutive study, in which we assessed the efficacy and safety of supra-choroidal (SC) Iluvien in the management of chronic diabetic macular edema (DME).

Sincerely,


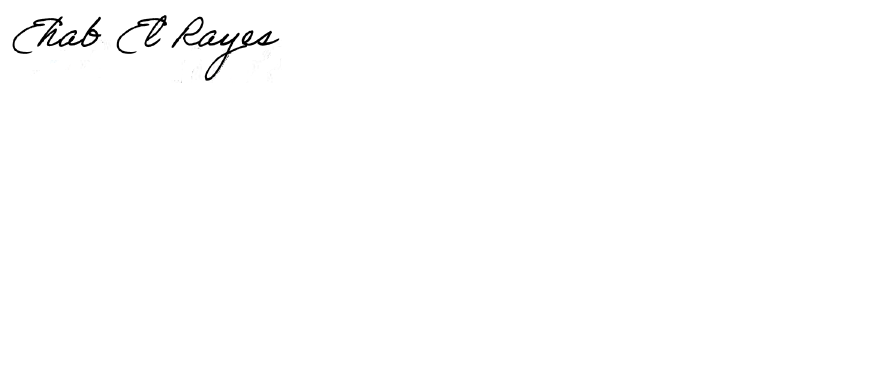


Ehab N. El Rayes, MD, PhD, FASRS
