# Supplementary material for: Visual function and retinal morphological changes after single suprachoroidal delivery of fluocinolone acetonide (Iluvien®) implant in eyes with chronic diabetic macular edema
Source: Int J Retina Vitreous. 2023 Mar 29;9:20. doi: 10.1186/s40942-023-00458-9 (PMC10053734; doi:10.1186/s40942-023-00458-9)
Supplement: Supplementary file 3 — Supplementary Material 3 [file 40942_2023_458_MOESM3_ESM.docx]

**Attention: Editorial Office – International Journal of Retina and Vitreous**

*Re: Manuscript - Visual function and retinal morphological changes after single suprachoroidal delivery of fluocinolone acetonide (Iluvien®) implant in eyes with chronic diabetic macular edema*

*Manuscript ID: IJRV-D-23-00017*

Response of the authors to comments made by reviewers

**I- Comments: Reviewer #1**

1) The SC placement of the drug lead to one implant being found in the intravitreal space. This means that 1 out of 12 eyes had migration of the implant into the invtravitreal space, meaning almost 10% had retinal breaks/ increased chance of RD. It would be helpful to compare the rate of these types of complications to prior studies which have looked at suprachoroidal drug delivery to see what rates of retina tears/detachments/intravitreal migration of drug occurred.

***Authors’ reply***

We would like to mention a number of points in reply to this comment,

1. For clarification, the study included 13 eyes of 13 patients. The patient who had migration of the implant into the vitreous cavity was excluded. This particular patient was the first one in the case series in which we employed the SC route of delivery of Iluvien. In our initial approach, we loaded the Iluvien implant into the Olive tip cannula followed by a bolus of viscoelastic. When the injection started, the bolus of viscoelastic displaced the implant into the suprachoroidal space. It is possible that the tip of the cannula penetrated the choroid and produced a retinal hole upon exit from the cannula. We learned from this complication to prime the Olive tip cannula first with viscoelastic before loading the implant and again another bolus of viscoelastic after loading it, so that upon starting the injection, the viscoelastic bolus exits first and opens the potential SC space, so that the dislodgement of the Iluvien implant and its entry into the SC space would be atraumatic.
2. We excluded the patient because the migration of the implant meant that the drug will be released intravitreally. This complication did not happen in the 12 eyes included in the statistical analysis.
3. The present study reported the possible benefits and risks of SC Illuvien in patients with chronic DME as a novel technique and as a base for future prospective comparative studies with a larger sample size.
4. The design of the present study is retrospective interventional non-comparative case series. This design is not an appropriate one to estimate the rate of incidence of any complication because answering this particular question requires different study designs firstly, to estimate the prevalence or incidence of a given complication and secondly, to determine the cause-effect relationship between a given complication and an intervention. Study designs as cohort, cross-sectional, case-control, and meta-analysis of randomized controlled clinical trials are required for this purpose. These designs usually contain a large number of patients in order to produce a correct statistical estimation of the incidence or prevalence of a particular complication, otherwise, any value in this regard would be an over- or under-estimation. For instance, one study that assessed the risk of RRD following intravitreal injections included 180671 injections in 12718 patients.
5. *Storey PP, Pancholy M, Wibbelsman TD, Obeid A, Su D, Borkar D, Garg S, Gupta O. Rhegmatogenous retinal detachment after intravitreal injection of anti-vascular endothelial growth factor. Ophthalmology 2019;126:1424-1431.*
6. *Mann CJ. Observational research methods. Research design II: cohort, cross-sectional, and case-control studies. Emergency Medicine Journal 2003;20:54-60.*

2) The Iluvien is a non-biodegradable implant. Does this surgical method leave within the suprachoroidal space remnants of an implant? What would the long-term follow up be for these remnants? Would they induce an inflammatory response, be an infectious nidus, or lead to necrosis in that area?

***Authors’ reply***

1. The delivery system on which Iluvien is mounted consists of polyimide tube, polyvinyl alcohol, silicone adhesive, and water for injection. These are inactive ingredients. They are inert and normally do not produce any of the complications that the reviewer mentioned. In fact, over the median one-year follow-up in the present study, we did not detect any of these complications.
2. Interestingly, after placement of the implant in the SC space we continued to detect its presence during follow-up visits on fundus biomicroscopy and on OCT scans for up to 18 months. In those patients who had follow-up periods of more than 18 months, we could not detect it by either method. It is possible that the pharmacological properties of the implant components change with the change of location in the eye due to different tissue interactions. Hence, the implant might undergo biodegradation when located in the SC space while remaining non-biodegradable in the vitreous. It is an interesting finding that worth exploring in future studies.

3) The authors state that this method lead to comparable improvements in vision. Were the baseline visual acuities similar between these patients and other studies evaluating the implant? In this current study, patients with chronic macular edema were included and that may mean that their visual acuity was also worse than the patients included in original studies.

***Authors’ reply***

Yes. The patients included in the present study had worse median baseline BCVA compared to other studies that discussed the use of Iluvien. The mean baseline BCVA in this study was 0.07 on the decimal scale compared to a mean of 52.9-54.7 ETDRS letters (approximately 0.25 decimal), 52.6 ETDRS letters (approximately 0.2 decimal), 48.8 ETDRS letters (approximately 0.2 decimal), and 51.9 ETDRS letters (approximately 0.2 decimal), 61.5 ETDRS letters (approximately 0.5 decimal) for the FAME, Medisoft audit, RESPOND, IRISS, and PALADIN studies, respectively.

4) The authors conclude that the SC administration of the drug is safer than the intravitreal administration, and I do not think that this claim can be made, given the migration of one implant into the intravitreal cavity and potential for retinal tears and detachment. I would instead focus on how the SC administration leads to less cataract and IOP rise if they believe this is true.

***Authors’ reply***

Please check our reply to the reviewer’s first comment.

**II - Comments: Reviewer #2**

1-      Line 146: "after previous treatment with at least 3 intravitreal injections of anti-VEGF agents or laser focal or grid photocoagulation." Authors need to add information about number of previous injections and/or laser treatment for each study eye.

***Authors’ reply***

- Two patients (17%) had received previously 6 and 8 intravitreal anti-VEGF injections, respectively. Four patients (33%) had received previously focal/grid laser photocoagulation combined and at least 1 intravitreal anti-VEGF injection. Six patients (50%) had received previously focal/grid laser photocoagulation solely. We added the requested information. Please check Results section, page 10, lines 204-208.

2-      In conclusion: " The SC approach for delivering Iluvien is a novel technique that is effective in improving visual function and reducing macular edema in patients with chronic DME. The SC route of administration helps reduce the incidence of steroid-induced cataracts and glaucoma and is non invasive compared to the intravitreal route." Authors may change conclusion statement. It is a small case series, it cannot confirm effectiveness of ILuvien in improving visual function and in reduction of cataract and glaucoma.

***Authors’ reply***

- The Conclusion statement has been re-phrased. Please check the Conclusion section, page 4, lines 82-84, and pages 12, and 13 lines 263-268.
